# Phytohormones 2020

**DOI:** 10.3390/biom12091305

**Published:** 2022-09-16

**Authors:** Guzel Kudoyarova

**Affiliations:** Laboratory of Plant Physiology, Ufa Institute of Biology, Ufa Federal Research Centre of the Russian Academy of Sciences, Pr. Octyabrya, 69, 450054 Ufa, Russia; guzel@anrb.ru

The hormonal system plays a decisive role in the control of plant growth and development. Alongside classical plant hormones (auxins, cytokinins, gibberellins, abscisic acid), the hormonal function is now attributed to jasmonates, salicylic acid, and brassinosteroids (BR). Plant hormones are capable of influencing vital processes, including plant growth and development, adaptation to the environment, and resistance to biotic and abiotic stresses and productivity. Accordingly, plant hormones are attractive tools for biotechnology aimed at improving plant performance in line with human needs. The synthesis of plant hormones by rhizosphere microorganisms is the basis for their capacity to promote plant growth as well as for the application of their preparations in crop production. Still, the success of the use of plant hormones depends on the knowledge of the mechanisms of their action. Advances in the study of plant hormones include the discovery of their receptors and cascades of hormonal signal transduction, identification of their target genes and those controlling hormonal metabolism and signaling, revealing cross-talk between hormones and their interaction with calcium, reactive oxygen species, and nitrogen oxide signaling.

The scope of this Special Issue is to provide a broad and updated overview on original research and review articles focused on the implication of plant hormones in plant growth and development, their adaptation to the environment and resistance to abiotic and biotic stresses (molecular, cellular, and whole plant aspects of the problems), cross-talk between plant hormones and their interaction with other signaling systems, and the importance of hormones for plant growth promotion by soil microorganisms.

The article of Andreeva et al. [1] reports on the cytokinin (CK)-regulated expression of *Arabidopsis* nuclear genes encoding plastid RNA polymerase-associated proteins (PAPs). CKs are known to regulate the biogenesis of chloroplasts, although, the underlying mechanisms are poorly understood. The mechanisms include the influence of nuclear genes on the expression of plastome. In this study, the authors demonstrated that CK induced the expression of twelve *Arabidopsis* PAP genes. The disruption of the genes in *pap1* and *pap6* mutants led to the abolishment of positive CK effect on the expression of nuclear genes for plastid transcription. However, the CK regulatory circuit in the mutants remained practically unperturbed. The knock-out of PAP genes resulted in cytokinin overproduction as a consequence of the up-regulation of the genes for CK synthesis.

One further article published in this issue addresses cytokinins [2]. It demonstrates the role of N-glucosylation in the complex process of CK homeostasis in plants. The presented results allow us to reconsider the widely held opinion about the irreversibility and inactivity of N7- and N9-glucosides of cytokinins. In this work, the authors demonstrate the specific physiological effects of CK N-glucosides in CK bioassays including their inhibitory effects on root development, and the activation of the CK signaling pathway visualized by the CK-responsive YFP reporter line. It reveals a considerable impact of N7- and N9-glucosides of cytokinins on the expression of CK-related genes in maize and their stimulatory effects on CK oxidase/dehydrogenase activity in oats.

The article of Adeyemi et al. [3] extends the cytokinin theme by exploring their applications in horticultural fruit crops. In this review, the authors briefly introduce the mode of action and general molecular biological effects of naturally occurring CKs before highlighting the great variability in the response of fruit crops to CK-based innovations. They present a comprehensive compilation of research linked to the application of CKs in non-model crop species at different phases of fruit production and management. The article demonstrates that current approaches using genomic-to-metabolomic analysis provide new insights into CK-derived actions that may serve as potential targets for improving crop-specific traits. CK molecular biology is discussed in the context of its present and future perspectives of the applications of CKs to fruits of horticultural significance.

The article of Hluska et al. [4] proposed the division of numerous representatives of cytokinins into two main categories based on their ability to act in the following two opposite ways: (1) a powerful but short-lived action of some cytokinins, such as trans-zeatin and isopentenyladenine, and (2) a subtle and enduring mode of action of other cytokinins, such as cis-zeatin. This review compared different cytokinin metabolites, their biosynthesis, translocation, and sensing to illustrate the different mechanisms behind the two CK strategies. This is applied to a plant developmental scale and, beyond plants, to interactions with organisms of other kingdoms, to highlight future research areas that can benefit the understanding of plant fitness and productivity.

The next [5] article of the issue addresses another plant hormone (abscisic acid, ABA), which plays an important role in plant growth and in response to abiotic stress factors. The authors assert that its accumulation in soil can negatively affect seed germination and plant growth. They studied the strain *Rhodococcus* sp. P1Y which was shown previously to assimilate ABA, thereby affecting its concentrations in plant roots. They identified the product of ABA decomposition by this bacterium as dehydrovomifoliol using different physico-chemical approaches. Based on the data obtained, it was concluded that the pathway of bacterial degradation and assimilation of ABA begins with a gradual shortening of the acyl part of the molecule.

The remaining seven articles in this issue are devoted to the role of hormones in plant resistance to abiotic and biotic stress factors. The review in [6] explored the action of ethylene as the master regulator of salinity stress responses. The article summarizes evidence supporting the capacity of ethylene to maintain the homeostasis of Na^+^/K^+^, nutrients, and reactive oxygen species (ROS) by inducing antioxidant defense in addition to elevating the assimilation of nitrates and sulfates. The review provides a comprehensive update on the prospects of ethylene signaling and its cross-talk with other phytohormones to regulate salinity stress tolerance in plants.

The next article reports on the involvement of jasmonic acid in the unfolded protein response (UPR) triggered by disturbance of the endoplasmic reticulum (ER stress) in tomato plants [7]. The article demonstrated that exogenous JA induced the transcript accumulation of UPR marker gene SlBiP, while the role of JA in ER stress sensing and signalling was confirmed in experiments with JA signalling mutant *jai1*. In addition, it was found that changes in hydrogen peroxide content, proteasomal activity, and lipid peroxidation are regulated by JA, while nitric oxide was not involved in ER stress and UPR signalling in leaves of tomato.

The article of Jin et al. [8] demonstrated the potential role of abscisic acid in the adaptation of *Onobrychis viciifolia* to extreme environmental conditions in the Qinghai–Tibetan plateau (QTP) as revealed by transcriptome analysis. Unlike most transcriptome studies performed in model experiments, the investigation was carried out under natural conditions. The research uncovered the up-regulation of genes potentially leading to changes in hormone homeostasis and signaling, particularly abscisic acid-related ones. The differentially expressed genes identified in this study represent candidate targets for improving the stress resistance of *O. viciifolia* grown in higher altitudes of the QTP, and can provide deep insights into the molecular mechanisms underlying the responses of this plant species to extreme environmental conditions.

Arkhipova et al. [9] reported on the effects of inoculating the culture medium of potato microplants grown in vitro with *Azospirillum brasilense* Sp245 or *Ochrobactrum cytisi* IPA7.2. The growth and hormone content of the plants were evaluated under stress-free conditions and under a water deficit imposed with polyethylene glycol (PEG 6000). Inoculation with either bacterium promoted growth in terms of leaf mass accumulation. The effects were associated with increased concentrations of auxin and cytokinin hormones in the leaves and stems and with the suppression of an increase in the leaf abscisic acid that PEG treatment otherwise promoted in the potato microplants. *O. cytisi* had a greater growth-stimulating effect than *A. brasilense* on stressed plants, while *A. brasilense* Sp245 was more effective in unstressed plants.

In the next paper [10], the authors characterized the molecular dynamics of chloroplast membranes that were isolated from wild-type and a BR-deficient barley mutant acclimated to low and high temperatures. The molecular dynamics of the membranes was investigated using electron paramagnetic resonance spectroscopy in both a hydrophilic and hydrophobic area of the membranes. The content of BR was determined, as well as chlorophylls, carotenoids and fatty acids. The chloroplast membranes of the BR-mutant had a higher degree of rigidification than the membranes of the wild type. The role of BR in regulating the molecular dynamics of the photosynthetic membranes is discussed against the background of an analysis of the photosynthetic pigments and fatty acid composition in the chloroplasts.

The root system is a prime responder to stress conditions in soil. Adventitious roots (AR) and lateral roots (LR) formation is affected by soil pollution, causing substantial root architecture changes. It is known that stress hormones such as jasmonates (Jas), ethylene (ET) and brassinosteroids (BRs) can regulate the division rate of cells in root meristems. The next article [11] presents a detailed overview of the crosstalk between JAs, ET, BRs, and the stress mediator nitric oxide (NO) in the auxin-induced formation of LRs and ARs with/without cadmium and arsenic, suggesting that stem cell activation may take part in developmental changes as a stress-avoidance-induced response. Interactions essential to achieving a balance between growth and adaptation to Cd and As soil pollution to ensure survival were reviewed here in the model species Arabidopsis and rice.

Crosstalk between ethylene and cytokinins has not been sufficiently studied as an aspect of plant immunity and was addressed in the present research [12]. The authors compared the expression of the genes responsible for hormonal metabolism and signaling in wheat cultivars differing in resistance to *Stagonospora nodorum* in response to their infection with fungal isolates, whose virulence depends on the presence of the necrotrophic effector SnTox3. Wheat susceptibility was shown to develop due to the decreased content of active cytokinins brought about by SnTox3-mediated activation of the ethylene signaling pathway. Exogenous zeatin enhanced wheat resistance against *S. nodorum* by inhibiting the ethylene signaling pathway and via the up-regulation of SA-dependent genes, while ethylene inhibited the triggering of the SA-dependent resistance mechanism, at least in part, by suppressing the cytokinin signaling pathway.

Nitric oxide (NO) is known to be involved in mediating hormonal signaling. The article of Gautam et al. [13] described how NO enhances photosynthetic nitrogen and sulfur-use efficiency and the activity of the ascorbate-glutathione cycle to reduce high-temperature-induced oxidative stress in rice plants.

## Data Availability

The data are available in the cited articles.
